# The Southern Surgical and Gynecological Association

**Published:** 1887-11

**Authors:** 


### SOCIETY NOTES.

### THE SOUTHERN SURGICAL AND GYNECOLOGICAL ASSOCIATION.

The meeting for the permanent organization of the Southern Surgical and Gynecological Association was held in Birmingham, Alabama, on the 12th of October, pursuant to a call by the Alabama Surgical and Gynecological Association, and was prompted by the many urgent requests of the most prominent physicians in the South, that the Alabama Association would extend its membership so as to include all the Southern States.

With this plan in view, the Secretary of the Association was authorized to correspond with a number of the leading physicians of each State and invite them to attend the annual session of the Alabama Surgical and Gynecological Association to consider the advisability of organizing such a society at that time.

Dr. H. N. Rosser was made temporary chairman, and Dr. W. E. B. Davis, secretary.

Membership was extended to the members of the Alabama Surgical and Gynecological Association, and to those who had responded fovorably to the secretary. The Constitution and By-

Laws differ from the State organization only in some minor particulars, made necessary by the enlarged scope of the new organization. The Association includes all the Southern States, and its meetings will be held on the second Tuesday of September of each year at such places as may be selected by the Association.

The following officers were elected:

Dr. W. D. Haggard, of Nashville, Tenn., President; Drs. R. D. Webb and J. W. Sears, both of Birmingham, First and Second Vice-Presidents, respectively; Dr. W. E. B. Davis, of Birmingham, Secretary; and Dr. H. P. Cochrane, of Birmingham, Treasurer.

The Judicial Couucil consists of the following:

Dr. Jno. S. Cain, Nashville, Tenn.; Dr. Hunter McGuire, Richmond, Va.; Dr. J. M. Taylor, Corinth, Miss.; Dr. DeSaussure Ford, Augusta, Ga.; Dr. R. A. Kinloch, Charleston, S. C.

Dr. W. F. Hyer, of Holly Springs, Miss.; was elected orator for the next annual session, which will be held in Birmingham.

THE STANDING COMMITTEES.

PUBLICATION COMMITTEE.—Drs. W. E. B. Davis, R. D. Webb and W. Locke Chew.

COMMITTEE OF ARRANGEMENTS.—Drs. J. D. S. D avi [illegible] Hughes, J. C. Dozier, J. B. Luckie and J. S. Gilespie.

The Publication Committee was authorized to invite known workers in Surgery and Gynecology to become members of the Association.

